# The Relationship between Fetal Abdominal Wall Thickness and Intrapartum Complications amongst Mothers with Pregestational Type 2 Diabetes

**DOI:** 10.1155/2021/5544599

**Published:** 2021-05-31

**Authors:** E. Paige Isabey, Christy L. Pylypjuk

**Affiliations:** ^1^Department of Obstetrics, Gynecology and Reproductive Sciences, University of Manitoba, Winnipeg, Canada R3A 1R9; ^2^Children's Hospital Research Institute of Manitoba, Winnipeg, Canada R3E 3P4

## Abstract

**Objectives:**

To evaluate the utility of fetal abdominal wall thickness (AWT) for predicting intrapartum complications amongst mothers with pregestational type 2 diabetes.

**Methods:**

This was a historical cohort study of pregnant mothers with pregestational type 2 diabetes delivering at a Canadian tertiary-care center between January 1, 2014, and December 31, 2018. Delivery records were reviewed to collect information about demographics and peripartum complications. Stored fetal ultrasound images from 36 weeks' gestation were reviewed to collect fetal biometry and postprocessing measurement of AWT performed in a standardized fashion by 2 blinded and independent observers. The relationship between fetal AWT was then correlated with risk of intrapartum complications including emergency Caesarean section (CS) and shoulder dystocia.

**Results:**

216 pregnant women with type 2 diabetes had planned vaginal deliveries and were eligible for inclusion. Mean maternal age was 31.3 years, and almost all were overweight or obese at the time of delivery (96.8%). Overall, the incidence of shoulder dystocia and emergency intrapartum CS was 7.4% and 17.6%, respectively. There was no difference in mean fetal AWT between those having a spontaneous vaginal delivery (8.2 mm (95% CI 7.9-8.5)) and those needing emergency intrapartum CS (8.1 mm (95% CI 7.4-8.8); *p* = 0.71) or shoulder dystocia (8.7 mm (95% CI 7.9-9.5); *p* = 0.23). There was strong interobserver correlation of AWT measurements (*r* = 0.838; *p* < 0.00001). The strongest association with intrapartum complications was birthweight (*p* = 0.003): with birthweight > 4000 grams, the relative risk of shoulder dystocia or CS is 2.75 (95% CI 1.74-4.36; *p* < 0.001).

**Conclusions:**

There was no obvious benefit of AWT measurement at 36 weeks for predicting shoulder dystocia or intrapartum CS amongst women with type 2 diabetes in our population. The strongest predictor of intrapartum complications remained birthweight, and so studies for improving estimation of fetal weight and evaluating the role of intrapartum ultrasound for predicting risk of delivery complications are still needed.

## 1. Introduction

Diabetes complicates ~5-7% of pregnancies worldwide, and numbers continue to increase in parallel with worsening rates of obesity [1, 2]. In our province, the prevalence of pregestational type 2 diabetes is amongst the highest in Canada, and with increasing rates, the number of affected pregnancies has also increased [1]. Pregestational diabetes increases the risk of perinatal complications for the mother, fetus, and newborn, including a higher risk of developing other medical complications of pregnancy such as preeclampsia. Another specific concern is the 4-5 times higher rate of stillbirth for mothers with pregestational type 2 diabetes, which has prompted increased efforts to improve antenatal surveillance and maternal glycemic control [3, 4]. Around the time of delivery, diabetes increases the risk of almost all peripartum complications of childbirth: induction of labor, Caesarean section (CS), operative vaginal delivery, high-degree lacerations, shoulder dystocia and related newborn injuries including asphyxia, postpartum hemorrhage, and prolonged hospital stay [3–5]. However, our ability to predict which patients with type 2 diabetes are most at risk of these intrapartum complications remains limited [3–8].

Shoulder dystocia complicates 1% of all births (even higher in those affected by diabetes) and can result in significant injury to newborns and mothers, and is also an independent risk factor of perinatal mortality [9]. Unfortunately, there is almost no way to further risk stratify these patients for individualized prediction of intrapartum shoulder dystocia or emergency intrapartum CS. Traditionally, fetal macrosomia has been the main risk factor of intrapartum complications: it is also the basis for several professional organizations recommending elective primary CS for large fetal size in pregnancies with or without diabetes [10]. However, studies from our center have highlighted the safety of vaginal delivery in the setting of fetal macrosomia, and thus, we have no current policy of elective primary CS for fetal macrosomia alone in the general population [11, 12]. Yet, local pregnant patients with uncontrolled diabetes plus fetal macrosomia are frequently induced around 36 to 37 weeks' gestation due to concerns about potential risk of stillbirth [1].

With advances in fetal ultrasound usage to predict intrapartum labor progress and success of vaginal delivery, there is the potential for its use to enhance prediction of specific intrapartum complications for women with pregestational diabetes as well. Fetal macrosomia is a major risk factor of intrapartum complications and birth trauma, including shoulder dystocia; however, there are concerns regarding performance of fetal ultrasound during late pregnancy to accurately predict postnatal weights [12–15]. Novel ultrasound techniques are being developed to improve antenatal prediction of macrosomia in order to prevent intrapartum birth complications: soft tissue measurements and other anthropometric markers as well as fetal volumes using three-dimensional ultrasound have all been suggested as ways to improve diagnosis of fetal overgrowth before delivery [16–18]. Cranial shape, ratio of abdominal-to-head circumferences, and biacromial measurements are proposed methods to enhance prediction of shoulder dystocia specifically [19–22]. Fetal abdominal wall thickness has also been proposed as a potential marker of shoulder dystocia or failed labor progress: however, the studies published thus far have been limited by small sample size and timing of ultrasound relative to delivery [21, 22]. To date, there have been some reports of an association between fetal abdominal wall thickness at midpregnancy ultrasound and prediction of gestational diabetes later in pregnancy; however, such findings need to be interpreted cautiously given the inherent difficulty of ensuring that women diagnosed with diabetes for the first time in pregnancy are truly those with gestational diabetes and not cases of undiagnosed type 2 diabetes which might otherwise explain the increased thickness of subcutaneous fetal fat [23]. In other preliminary work from our group, there does appear to be a difference in the abdominal wall thickness of fetuses exposed to pregestational type 2 diabetes: fetuses exposed to diabetes in utero have significantly thicker subcutaneous abdominal wall fat than those born to healthy controls [24]. The goal of this study was to evaluate the utility of fetal abdominal wall thickness (AWT) in the third trimester for predicting intrapartum complications amongst mothers with known pregestational type 2 diabetes.

## 2. Materials and Methods

This was a historical cohort study conducted at the Health Sciences Centre Women's Hospital in Winnipeg, Canada, over a 5-year period between January 1, 2014, and December 31, 2018. This tertiary-care hospital serves as one of two regional referral sites for a total population of 1.3 million inhabitants and a geographic region which includes urban, rural, and northern/remote communities: it also represents the highest concentration of diabetes in pregnancy in the region. There are approximately 5000 to 5500 deliveries per year at the study hospital and over 10,000 ultrasounds performed within its Fetal Assessment Unit annually. Research ethics approval was obtained from the University of Manitoba Health Research Ethics Board. Because this project was retrospective in nature and did not require any direct patient contact, individual consents were not required by our institution.

All pregnant patients with a diagnosis of pregestational type 2 diabetes and delivering at the study hospital during the 5-year period were eligible for inclusion. Potential study subjects were identified using delivery record books and the diagnosis of type 2 diabetes cross-validated with the maternal diagnosis entered in the stored fetal assessment record. Cases of multiples, congenital anomalies, planned postnatal palliation, planned delivery by Caesarean section, and those delivering prior to 36 weeks were excluded. Cases were also excluded if they did not have stored fetal ultrasound images from a 35- to 36-week scan, noting that it is the local standard of care to perform a fetal assessment scan for all patients with type 2 diabetes during that time period.

**Figure 1:**
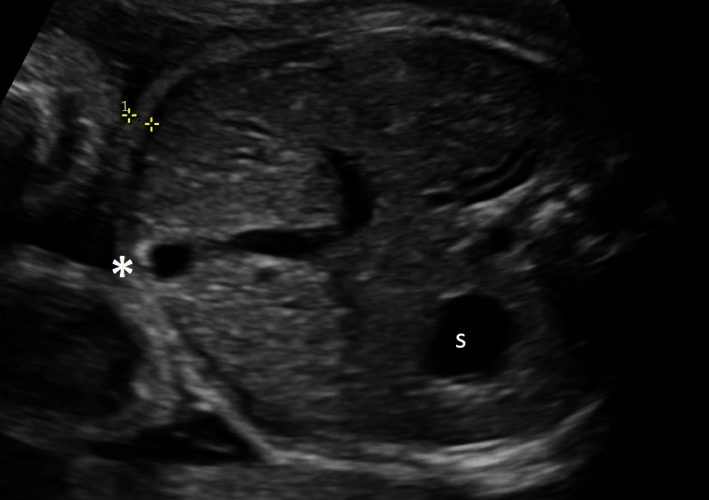
Anterior abdominal wall thickness measurement (calipers) as obtained from the standard abdominal circumference view. S = stomach bubble; ^∗^area of cord insertion near origin of portal umbilical vein complex.

Hand searches of delivery record books were performed by experienced research personnel to identify potential cases of pregestational type 2 diabetes and information regarding basic maternal demographics, pregnancy and delivery information, and early postnatal outcomes abstracted using standardized data collection sheets. Postprocessing review of stored ultrasound images and fetal assessment reports was also performed to obtain data about fetal biometry and measurements of abdominal wall thickness. Abdominal wall thickness measurements were performed in a standardized fashion as described by Higgins et al. in 2008 [25] and utilize the standard, transverse axial section view of the fetal abdomen commonly obtained for measurement of the abdominal circumference [26]: in this plane and at the level of the stomach bubble and portal umbilical venous complex, the thickest area of the subcutaneous layer in the near-field anterior abdominal wall within 45 degrees of the cord insertion is measured (Figure 1). Written consent was obtained by the individual patient for use of this ultrasound image. A second blinded observer performed repeated measurement of fetal abdominal wall thickness in a random selection of 25% of cases to ensure interobserver reliability. Where multiple scans were performed during this time frame, the scan closest to delivery was chosen and used to obtain the measurements of interest. Biometry and abdominal wall thickness were then correlated with intrapartum complications (shoulder dystocia and emergency intrapartum Caesarean section). Macrosomia in the fetus was defined as estimated fetal weight above the 90^th^ percentile for gestational age on fetal growth curves standardly used in our unit; neonatal macrosomia was defined separately as birthweight above 4500 grams and as greater than the 90^th^ percentile at birth by the newborn growth curves used locally [26–28]. All patients in the cohort had adequate follow-up until delivery.

Statistical analysis was performed using Stata v.14.2 (StataCorp LLC, College Station, TX) software, with a *p* value less than 0.05 used to denote statistical significance. Continuous variables were presented as means with 95% confidence intervals (or standard deviations) if normally distributed or as medians with interquartile ranges if nonparametrically distributed. Dichotomous and categorical variables were described as proportions. Student's *t*-, chi-square, Wilcoxon rank-sum, Kruskal-Wallis, and analysis of variance tests were used to compare outcomes between groups depending on data type and distribution. Linear regression analyses were performed to evaluate the relationship between fetal ultrasound measurements of abdominal wall thickness and abdominal wall circumference, as well as estimated fetal weight: logistic regression was then used to evaluate the crude odds of intrapartum complications by individual ultrasound measurements and birthweight (given the inherent error of estimated fetal weight measurements [ref]). The Spearman correlation coefficient was used to evaluate interobserver reliability of abdominal wall thickness measurements.

## 3. Results and Discussion

There were 216 patients that met study criteria and included in the analysis. In our cohort, pregnant women with pregestational type 2 diabetes had a mean age in years of 31.3 (SD 6.5) and most were multiparas (77.6%) (Table 1). The mean body mass index (BMI) at delivery was high at 36.6 kg/m^2^: only 3.2% of the entire cohort had a normal BMI and 96.8% were considered overweight or obese, including one-third that were categorized as class 3 obesity with a BMI ≥ 40 kg/m^2^. 34.4% of these pregnancies were complicated by additional medical conditions, including 18.8% with hypertensive disorders of pregnancy, although there was only 1 documented case of preeclampsia. According to fetal ultrasound findings antenatally, 21.8% of cases were suspected to have fetal growth abnormalities prior to delivery: 21.3% were diagnosed with fetal macrosomia > 90^th^ percentile for gestational age along with 0.5% diagnosed with fetal growth restriction < 10^th^ percentile for gestational age (Table 1). The mean abdominal wall thickness of fetuses exposed to pregestational type 2 diabetes was 8.2 mm (95% CI 8.0-8.4).

**Figure 2:**
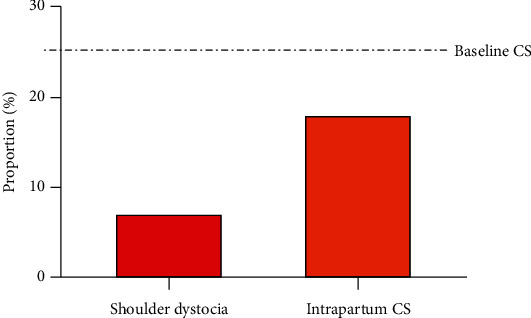
Proportion of deliveries complicated by shoulder dystocia and intrapartum Caesarean section (CS), compared to the baseline CS risk in the population (25.4%) [20].

The majority of patients in our cohort were induced (81.9%) (Table 1). 32.1% required some form of cervical ripening, either by chemical or mechanical means (Table 1). Almost half of patients (41.6%) required oxytocin at some point during the process of induction. Most patients had spontaneous vaginal deliveries (71.3%), whereas 9.7% required operative vaginal deliveries and another 19% had CS deliveries (with an overall prevalence of “emergency” intrapartum CS equal to 17.6%) (Table 1 and Figure 2). 7.4% of vaginal deliveries were complicated by shoulder dystocia. The median gestational age at delivery was 37 + 1 weeks' gestation [IQR 36 + 0 to 38 + 3]. Apgar scores were 8 [IQR 6 to 9] and 9 [IQR 9 to 9] at one and five minutes, respectively: fewer than 3% of deliveries were complicated by a 5-minute Apgar score less than 7. About half of the newborns in the cohort were female. Mean birthweight was 3529.8 grams (95% CI 3440-3620), and only 6% of deliveries were designated as macrosomic at birth using the definition of >4500 grams: however, by using greater than the 90^th^ percentile for gestational age to define macrosomia, 32.9% of newborns in the cohort were considered macrosomic at birth.

Regarding perinatal characteristics differentiating pregnancies with and without delivery complications, cases with shoulder dystocia or intrapartum CS had significantly higher BMIs than those with spontaneous vaginal deliveries (*p* = 0.026) (Table 2). Pregnancies resulting in emergency intrapartum CS were more likely to have other comorbid medical complications but a trend towards fewer inductions of labor. One-minute Apgar scores were significantly lower amongst those deliveries complicated by shoulder dystocia (*p* = 0.013), but there was no difference in 5-minute Apgar scores between the three groups (*p* = 0.788) (Table 2). There was no significant difference in mean fetal abdominal wall thickness between those having spontaneous vaginal deliveries (8.2 mm (95% CI 7.9-8.5)) and those requiring emergency intrapartum CS (8.1 mm (95% CI 7.4-8.8); *p* = 0.71) or those deliveries complicated by shoulder dystocia (8.7 mm (95% CI 7.9-9.5); *p* = 0.23) (Figure 2). There was moderate positive correlation between abdominal circumference and abdominal wall thickness (*r* = 0.548; *p* < 0.0001) and strong interobserver correlation of AWT measurements (*r* = 0.838; *p* < 0.00001). The strongest association with intrapartum complications was birthweight (*p* = 0.003): with birthweights > 4000 grams, the relative risk of shoulder dystocia or CS is 2.75 (95% CI 1.74-4.36; *p* < 0.001).

## 4. Discussion

**Figure 3:**
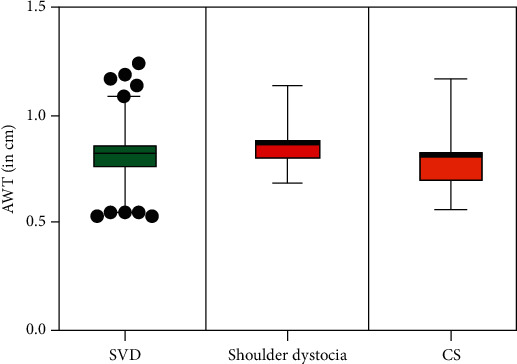
Abdominal wall thickness (AWT) by intrapartum outcome (spontaneous vaginal delivery (SVD), shoulder dystocia, and Caesarean section (CS)).

Incidence of pregestational type 2 diabetes mellitus in pregnancy is steadily increasing across the world and along with it the associated antenatal and intrapartum complications. As evidenced by our study, the frequency of shoulder dystocia in our cohort of women with pregestational type 2 diabetes of 7.4% is much higher compared to that of the general obstetric population of 0.2-3.0% [9]. However, the risk of CS amongst women with pregestational type 2 diabetes was lower than the baseline population risk of CS at our center (17.6% versus 25.4%) [29]: this finding might be reflective of local practice patterns whereby pregnancies complicated by poorly controlled diabetes plus fetal macrosomia are routinely induced around 36-37 weeks' gestation due to concerns about stillbirth risk [1, 6, 10] (Figure 3). In addition to the lower CS rate, the overall risk of immediate newborn complications was also low in this cohort: fewer than 3% of newborns had a 5-minute Apgar less than 7 (incorporated as a proxy for fetal asphyxia), and there were no cases of intrapartum birth injuries or fractures amongst these neonates. This particular pregnancy cohort (women with type 2 diabetes) was chosen as the study group of interest given the existing evidence regarding frequency of intrapartum complications and an assumption that if there was a true association between fetal AWT and shoulder dystocia or intrapartum complication, the best chance of finding a relationship would be in this restricted high-risk population: it also eliminated any possibility of bias that might occur when including women diagnosed with gestational diabetes who may in fact represent women with previously undiagnosed type 2 diabetes.

While our study results did not show any benefit of fetal AWT measurement at 36 weeks' gestation in the prediction of shoulder dystocia or emergency intrapartum Caesarean section, this again could be reflective of our local practice of inducing women with poorly controlled type 2 diabetes between 36 to 37 weeks' gestational age: most AWT measurements were taken within one week of delivery, but AWT might be more significant if taken upon admission to hospital in labor and/or if interpreted relative to other measurements of fetal biometry (i.e., head circumference) instead of as an isolated marker. Because this study is unable to determine if fetal AWT might be influential in centers without such a high frequency of late preterm inductions for women with poorly controlled pregestational type 2 diabetes, additional studies are needed to explore AWT and other potential ultrasound markers to predict risk or success of a vaginal delivery in pregnancies both with and without diabetes: concurrently, evaluation of policies regarding timing of induction of labor which directly compare the risk/benefits of late preterm delivery on stillbirth prevention versus neonatal sequelae is also needed to ensure optimal care for pregnant women with diabetes. There is heightened interest for use of intrapartum ultrasound particularly since the inception of new professional guidelines for use of ultrasound on the labor floor as well as individual studies which have highlighted the utility of ultrasound to evaluate likelihood of successful vaginal delivery [30–33]. In our cohort of patients with high rates of labor induction, the strongest relationship between intrapartum complications (shoulder dystocia or emergency intrapartum CS) remained birthweight. Those deliveries requiring emergency intrapartum CS tended to have lower rates of induction of labor compared to those resulting in spontaneous vaginal delivery, thus dispelling potential concerns about a risk of CS due to induction of labor which is consistent with the literature. It was notable that fetal ultrasound in our center tended to underdiagnose fetal macrosomia compared to postnatal diagnosis using birthweights over the 90^th^ percentile for gestational age: this finding was consistent with another preliminary work by our team with a similar population and likely impacted by the high rates of morbid obesity in this group as well as the inherent limitations of fetal ultrasound to accurately predict newborn weight during late pregnancy [5, 15]. Diagnostic thresholds that use a cut-off of 4500 grams to designate macrosomia in the newborn are also likely to underestimate the frequency of fetal overgrowth in this population or for other populations where delivery before term is undertaken [10]. The need to explore improved models of estimated fetal weight or novel markers of fetal body composition, particularly amongst women with pregestational type 2 diabetes, is necessary to better refine risk prediction of intrapartum complications in this high-risk group [32, 34, 35].

The global diabetes epidemic closely parallels trends in rising obesity, and the rates of obesity in this study population cannot be understated: with almost 97% of pregnant women with pregestational type 2 diabetes in our cohort classified as overweight or obese at the time of delivery, enhanced efforts to improve preconceptional health and weight management as well as strategies to address appropriate weight gain during pregnancy are urgently needed. There is also evidence that the current COVID-19 pandemic, particularly the restrictions on daily activities, has further exacerbated problems of inactivity and weight gain in pregnancy [36]. Given what is known in the literature about the effects of multiparity on weight gain and likelihood of long-term obesity and health risks following postpartum weight retention, the fact that two-thirds of mothers in our cohort were multiparas may have been contributory to our findings of high BMI [37–40]. In our study, women with higher BMIs were significantly more likely to have intrapartum complications (shoulder dystocia and emergency intrapartum CS). The increased risk of shoulder dystocia with maternal obesity is consistent with what is described in the literature, as is the heightened risk of CS: however, we are unable to determine with certainty if the frequency of emergency intrapartum CS in our population was exclusively driven by maternal obesity leading to intrapartum dystocia or failure of labor progress or if there is confounding by indication—could obstetricians have a lower threshold for recommending intrapartum CS earlier or more frequently in women with type 2 diabetes and high BMIs due to concerns about an inability to perform a crash CS if one became indicated? Both diabetes and obesity are associated with hypertensive disorders of pregnancy, and almost 1 in 5 women in our cohort had this complication of pregnancy as well. It was notable that there was only one case of preeclampsia diagnosed in this high-risk group; however, this might also reflect a potential impact of earlier induction of labor on reducing the development of preeclampsia in this high-risk group. In modern maternity care, strategies regarding appropriate weight gain and postpartum weight loss are counselled and managed at the individual patient level, although this study highlights the importance of considering broader public health policies to improve BMI amongst reproductive age women and particularly those with comorbidities such as diabetes [41–43]. With evidence that adherence to a Mediterranean diet during COVID-19 is protective against gestational diabetes during the pandemic and other virtual weight loss technologies are effective at supporting postpartum weight loss, these tools offer innovative solutions for mothers of young children and newborns, even through times of physical distancing and pandemic quarantines [44–46]. At a minimum, achievement of a healthy BMI for women with pregestational diabetes specifically will reduce diabetes-related morbidity in addition to improving perinatal outcomes by reducing intrapartum complications [39, 42, 43].

The relationship between fetal AWT and long-term health of offspring remains unknown. With evidence to support increasing prevalence and disease severity of type 2 diabetes with each successive generation affected [46–48], there is question as to whether or not a thicker fetal subcutaneous fat layer might represent an early marker of future metabolic disease. Overall, fetuses in our study had thicker subcutaneous fat layers than described in other studies (8.2 mm at 35 to 36 weeks versus 5.4 mm at 35 to 39 weeks in the Higgins study) [25]: this difference may be related to the restriction of our study population to only those mothers with confirmed pregestational type 2 diabetes or it may be a consequence of a poorer underlying maternal metabolic environment of mothers in our cohort including higher rates of morbid obesity. However, with an AWT of less than 4 mm proposed as the “normal” cut-off for fetuses between 36 and 38 weeks' gestational age, the subcutaneous fat thickness of offspring in this cohort remains considerably higher by comparison as well [23]. While there was not either an obvious relationship between fetal AWT and intrapartum asphyxia or birth trauma, we were underpowered to comment on these risks definitively given the rarity of these complications in our study population. Ongoing work is needed to elucidate any potential linkage between subcutaneous fat thickness in offspring and possible fetal origins of future metabolic disease, particularly given the worsening prevalence of childhood-onset diabetes in our health region and around the world [1, 47, 49, 50]: if a relationship between fetal AWT and long-term metabolic disease exists, this could offer considerable lead time and an opportunity for interventions to improve health and reduce chronic diseases in children exposed to maternal type 2 diabetes in utero.

Benefits of this study include a large sample size and incorporation of a novel fetal biometric measurement (AWT) using existing ultrasound images taken at the time of routine 36-week ultrasound. With excellent interobserver reliability, our study showed that fetal AWT measurement can easily and practically be incorporated at the time of third trimester ultrasound and using the standard images already obtained during measurement of the fetal abdominal circumference, without requiring any additional healthcare resources or costs. Since we restricted our study population to women with known, pregestational type 2 diabetes, we ensured a universal exposure of the entire study population: as previously mentioned, one risk of including all patients with diabetes in pregnancy without restriction is that it is difficult to know with certainty if women diagnosed with gestational diabetes have true hyperglycemia with onset only in pregnancy versus misclassified women with previously undiagnosed type 2 diabetes. As a retrospective cohort study, there are inherent limitations such as information and misclassification bias and missing data. We were also unable to evaluate the influence of individual-level glycemic control or ethnicity on fetal abdominal wall thickness. Future research is needed to evaluate the role of additional ultrasound predictors of intrapartum complications within the general obstetric population beyond diabetes, including fetal AWT at later gestational ages closer to delivery, and considering the relative influence of AWT combined with other fetal measurements (i.e., head circumference or biparietal diameter) for intrapartum risk stratification. The relationship between fetal AWT and long-term health of offspring exposed to maternal type 2 diabetes in utero also remains unknown.

## 5. Conclusions

There was no obvious benefit of adding fetal AWT measurement at 36 weeks for predicting shoulder dystocia or intrapartum CS in a population of women with pregestational type 2 diabetes in a setting where routine induction of labor is undertaken for those with poor glycemic control and high risk of stillbirth. The strongest predictor of intrapartum complication remains birthweight, and so studies evaluating improved methods for estimating fetal size (weight) and the role of intrapartum ultrasound for enhancing prediction of delivery complications are still needed. The potential relationship between fetal AWT and long-term health in offspring also requires further investigation.

## Figures and Tables

**Table 1:** Maternal characteristics and peripartum outcomes associated with pregnancies affected by pregestational type 2 diabetes.

Variable of interest	Total cohort (*n* = 216)
Maternal age in years, mean (SD)	31.3 (6.5)
Gravidity, median [IQR]	3 [2 to 6]
Gravidity > 1 (%)	86.1%
Parity, median [IQR]	2 [1, 3]
Parity > 0 (%)	77.6%
Body mass index^a^, mean (SD)	36.6 kg/m^2^
BMI < 18.5, underweight (%)	0
BMI 18.5-24.9, normal (%)	3.2%
BMI > 25–29.9, overweight (%)	13.7%
BMI 30-34.9, class 1 obesity (%)	25.9%
BMI 35-39.9, class 2 obesity (%)	24.9%
BMI > / = 40, class 3 obesity (%)	32.3%
Other medical complications of pregnancy (%)	34.4%
Hypertensive disorders	18.8%
Other maternal complications	11.6%
Fetal growth abnormalities on US (%)	21.8%
Macrosomia > 90^th^ %ile for GA	21.3%
IUGR < 10^th^ %ile for GA	0.5%
Induction of labor (%)	81.9%
Prostaglandin gel	13.1%
Prostaglandin insert	14.9%
Foley catheter or cervical ripening balloon	4.1%
Artificial rupture of membranes	21.6%
Oxytocin	46.3%
Gestational age at delivery, median [IQR]	37 + 1 [36 + 0 to 38 + 3]
Mode of delivery (%)	
Spontaneous vaginal delivery	71.3%
Assisted vaginal delivery	9.7%
Caesarean section	19%
1 min Apgar	8 [6, 9]
5 min Apgar	9 [9, 9]
5 min Apgar < 7 (%)	2.8%
Birthweight in grams, mean (SD)	3529.8 (655.3)
>4500 grams (%)	6.0%
>90^th^ %ile for GA (%)	32.9%
Female fetus (%)	52.1%

Notes: ^a^calculated for *n* = 189 with available BMI data.

**Table 2:** Perinatal characteristics and birth outcomes associated with intrapartum complications.

	Spontaneous vaginal delivery (*n* = 154)	Shoulder dystocia (*n* = 13)	Caesarean section (*n* = 38)	*p* value
Multiparous (%)	81.6%	76.9%	73.8%	0.511
Body mass index^a^, mean (SD)	36.1 (7.3)	39.3 (7.7)	39.3 (6.6)	0.026
BMI < 18.5, underweight (%)	0	0	0	—
BMI 18.5-24.9, normal (%)	3.7%	0	0	—
BMI > 25–29.9, overweight (%)	17%	0	5.9%	—
BMI 30-34.9, class 1 obesity (%)	25.9%	0	17.6%	—
BMI 35-39.9, class 2 obesity (%)	23.7%	41.7%	26.5%	0.470
BMI > / = 40, class 3 obesity (%)	29.7%	58.3%	50%	0.011
Other medical complications of pregnancy	16%	15.4%	32.6%	0.092
Hypertensive disorders	15.8%	15.4%	16.3%	0.713
Other maternal conditions	8.2%	0	23.2%	—
Induction of labor (%)	91.4%	91.7%	79.3%	0.076
Gestational age at delivery, median [IQR]	37 [36 to 38]	37 [36 to 38]	37 [36 to 38]	0.899
1 min Apgar	8 [6.5 to 9]	6 [6 to 7]	8 [4.5 to 9]	0.013
5 min Apgar	9 [9 to 9]	9 [9 to 9]	9 [9 to 9]	0.788
Birthweight in grams, mean (SD)	3469.4 (627.38)	3992.9	3679.6 (805.3)	0.008
Birthweight > 4500 grams (%)	5.1%	(276.8) 30.8%	10.5%	0.001
Birthweight > 90 %ile for GA (%)	43.4%	84.6%	71.1%	0.0004
Female fetus (%)	55.7%	38.5%	44.2%	0.110

Notes: ^a^calculated for *n* = 189 with available BMI data.

## Data Availability

Data may be available upon reasonable request.
